# Can Growth of *Nannochloropsis oculata* under Modulated Stress Enhance Its Lipid-Associated Biological Properties?

**DOI:** 10.3390/md20120737

**Published:** 2022-11-24

**Authors:** Sérgio C. Sousa, Manuela Machado, Ana C. Freitas, Ana M. Gomes, Ana P. Carvalho

**Affiliations:** 1 Escola Superior de Biotecnologia, CBQF—Centro de Biotecnologia e Química Fina—Laboratório Associado, Universidade Católica Portuguesa, Rua Diogo Botelho 1327, 4196-005 Porto, Portugal; 2 REQUIMTE/LAQV—Instituto Superior de Engenharia, Instituto Politécnico do Porto, Rua Dr. António Bernardino de Almeida 431, 4200-072 Porto, Portugal

**Keywords:** polyunsaturated fatty acids, eicosapentaenoic acid, antioxidant capacity, metabolic activity, adipolysis, steatosis, anti-inflammatory activity, cytokines

## Abstract

*Nannochloropsis oculata* is well-recognized as a potential microalgal source of valuable compounds such as polyunsaturated fatty acids, particularly, eicosapentaenoic acid (EPA). The content and profile of these lipids is highly dependent on the growth conditions and can, therefore, be tailored through modulation of the growth parameters, specifically, temperature. Moreover, biological activities are composition dependent. In the present work, lipid extracts obtained from *N*. *oculata*, grown under constant temperature and under modulated temperature stress (to increase EPA content; Str) were characterized by GC-FID and several bioactivities were evaluated, namely, antioxidant (L-ORAC_FL_), cytotoxic (MTT), adipolytic, anti-hepatic lipid accumulation (steatosis), and anti-inflammatory properties. Both extracts exhibited antioxidant activity (c.a. 49 µmol Trolox_equivalent_/mg_extract_) and the absence of toxicity (up to 800 µg/mL) toward colon and hepatic cells, adipocytes, and macrophages. They also induced adipolysis and the inhibition of triglycerides hepatic accumulation, with a higher impact from Str. In addition, anti-inflammatory activity was observed in the lipopolysaccharide-induced inflammation of macrophages in the presence of either extract, since lower levels of pro-inflammatory interleukin-6 and interferon-β were obtained, specifically by Str. The results presented herein revealed that modulated temperature stress may enhance the health effects of *N. oculata* lipid extracts, which may be safely utilized to formulate novel food products.

## 1. Introduction

Microalgae are widely recognized as potential sources of a vast array of valuable compounds, such as lipids, polysaccharides and proteins, among others, which present distinct biological (e.g., antioxidant, anti-inflammatory and antimicrobial) activities [1,2,3,4,5]. Due to this potential, microalgae present a high versatility for their utilization in developing products in distinct areas such as the food and feed, pharmaceutical, cosmetic, and biofuels industries [4,6,7,8,9]. *Nannochloropsis oculata* is a microalgae which possesses a significant amount of eicosapentaenoic acid (EPA) [10,11], an omega-3 polyunsaturated fatty acid (ω-3 PUFA) which belongs to a class of fatty acids known for the potential health benefits that their consumption may promote, such as the reduction in inflammation and insulin resistance, the prevention of cardiovascular disease (CVD), and improvement of brain functions, among others [12,13]. Omega-3 PUFA decrease the levels of plasma triglycerides, hence reducing the risk of CVD in patients with hypertriglyceridemia [14]. They also reduce the production of pro-inflammatory cytokines, such as interleukin 6 (IL-6) and tumor necrosis factor alpha (TNF-α) [12], which may help patients with autoimmune disease. Obesity and diabetes are two other health related problems which may be positively impacted, due to decreased cholesterol biosynthesis and obesity-induced insulin resistance induced by ω-3 PUFA consumption [12]. Microalgae composition (contents and profiles), including that of fatty acids, is intrinsically dependent on growth conditions, such as temperature, light, nutrient availability, and salinity, all of which impact microalgal metabolism and, consequently, composition [6,11,15]. Hence, it is possible to modulate growth conditions in order to modify the production of a specific compound (or type of compound). For example, PUFA content can be increased by decreasing temperature [11,15,16]. Since biological activities are composition dependent, the growth conditions of microalgae also have an impact on these activities. *Nannochloropsis oculata* lipophilic extracts have been reported to comprise several distinct compounds. Fatty acids are the main compounds described as being present in lipid extracts of the microalga [17,18,19], although other compounds such as sterols [20] and carotenoids [21] have also been determined to be found therein. Moreover, studies have showed relevant biological activities associated with *N*. *oculata* lipophilic extracts, such as antioxidant capacity and anti-inflammatory and anti-cancer activities [20,21,22].

The aim of the present work was to determine the impact of *N*. *oculata* growth under EPA-increasing modulated stress in the composition of a lipophilic extract (fatty acid, sterol, and pigment profiles) and assess its repercussions regarding lipid-associated antioxidant capacity and biological properties, namely, cytotoxic (inhibition of cell metabolism), adipolytic, anti-hepatic lipid accumulation, and anti-inflammatory properties.

## 2. Results and Discussion

Microalgae are known sources of many different compounds, such as fatty acids, sterols, pigments, carbohydrates, and proteins, among others. It has been found that some microalgae compounds can present a panoply of different interesting biological activities, ranging from antibacterial, antifungal, and antiviral activities, to immunostimulatory and anti-inflammatory activities [23]. The biological activity of a microalgae extract will be intrinsically dependent on its composition, which in turn, will be dependent on biomass composition and extraction methodology. The composition of a given extract will result from the extraction methodology and solvents used—which will determine the type and amount of compounds that are extracted—and from growth conditions—which will determine biomass composition. The manipulation of extraction methods and growth parameters can modulate the composition of microalgae extracts and, consequently, their biological activities.

### 2.1. Lipid Extracts Characterization

#### 2.1.1. Fatty Acid Profiles

Fatty acid profiles (Table 1) showed that extracts from both *N. oculata* cultures were mainly composed of C16:0 (palmitic acid), C16:1 c9 (palmitoleic acid), and C20:5 ω3 (eicosapentaenoic acid; EPA), which together represented 80 and 77% of total fatty acids (TFA), in Ctrl and Str extracts, respectively. The extract from *N. oculata* grown under normal conditions presented higher palmitic and palmitoleic acid contents than did the stressed culture (159.41 and 158.71 mg/g_extract_, respectively, vs. 124.13 and 132.38 mg/g_extract_ in Str extract), while EPA was, as expected, higher in the extract from the stressed culture (51.07 vs. 45.25 mg/g_extract_, in Str and Ctrl, respectively, corresponding to 12.8 and 9.9% of TFA). The Str extract also possessed higher total ω3 and lower total ω6 fatty acids contents than the Ctrl, and the total amounts of polyunsaturated fatty acids (∑PUFA) were not significantly different (*p* > 0.05) between the extracts. The extract obtained from the Ctrl culture contained higher (*p* < 0.05) total saturated and monounsaturated fatty acids (∑SFA and ∑MUFA) contents and TFA. In addition to the fatty acid profiles, two indices and one ratio pertaining to lipid quality, which are indicative of the nutritional quality of the lipids, were also calculated. Concerning those indicators, the Str presented lower (*p* < 0.05) AI and TI indices than the Ctrl (0.81 and 0.58, compared with 0.86 and 0.73), and a higher (*p* < 0.05) HH ratio (0.71 vs. 0.53).

Fatty acid profiles obtained from both extracts were in accordance with the findings of other studies concerning *Nannochloropsis*, and more specifically, *N*. *oculata*. Zhang et al. (2020), studying a two-step procedure to obtain bioactive compounds from *N*. *oculata*, found the predominant fatty acids to be palmitic (25–29%) and palmitoleic acids (28–32%), along with EPA (24–31%) [4]. Shene et al. (2016) explored the production of EPA by *N*. *oculata*, also determining those fatty acids as the ones which presented the highest amounts, always representing over 72% (72–79%) of total fatty acids, and varying the predominance among them according to different growth conditions [24]. Similar fatty acid compositions of *N*. *oculata* has also been reported by other authors [11,17,25,26] for several years [27,28]. The relatively low EPA and PUFA percentages obtained in the present study were a consequence of the growth phase of the biomass when it was collected. It is known that fatty acid composition varies throughout growth cycle, and the percentages of those fatty acids, although increasing in the early growth stages, decrease with culture age, while SFA and MUFA present an opposite behavior [28,29]. The point in time at which the cultures were collected was chosen so that the amount of EPA extracted would be the highest, although in terms of relative percentages in the extract, the EPA and PUFA percentages were low. Regarding the differences found in those fatty acids between the Ctrl and Str, the higher EPA and PUFA percentages found in the Str extract were in accordance with the results reported in our previous work [30], as well as those of other authors [11,31,32,33], in which microalgae cultures exposed to decreases in temperature increased the amounts of such fatty acids, as a (coping) mechanism to increase membrane fluidity, which is reduced when temperature decreases.

The nutritional value of the extracts was, as previously mentioned, assessed through the determination of two indices and one ratio, which ultimately translate the potential to impact human health, as different types, groups, or specific fatty acids can have a beneficial or detrimental effect on the organism. The correlation between the percentages of specific groups of lipids and the corresponding qualitative aspects can provide indication about the protective or deleterious potential towards coronary heart disease [34].

The atherogenic index (AI) is determined through the ratio of specific SFA and specific unsaturated fatty acids, since some SFA can increase the levels of blood cholesterol (pro-atherogenic), and anti-atherogenic effects are associated with MUFA and PUFA [35]. The thrombogenic index (TI) assesses the tendency for formation of blood clots in blood vessels [35], associating thrombogenic SFA with MUFA and ω3 and ω6 PUFA, which are anti-thrombogenic [11]. Therefore, these indices provide some indication regarding the potential of the extracts towards the increase of atheroma and or/thrombus formation [11], or to protect against coronary heart disease [36]. AI and TI values are inversely related with the lipid nutritional quality [34]. The hypocholesterolemic/hypercholesterolemic (HH) ratio is related to the impact of specific fatty acids on cholesterol metabolism, as it is known that unsaturated fatty acids (MUFA and PUFA) decrease serum cholesterol, while some SFAs, namely, lauric (C12:0), myristic (C14:0), and palmitic acids, raise serum cholesterol [34,35,36].

The nutritional quality of the extracts obtained herein was not as positive as the findings of other researchers, although an improvement could be observed in the Str when compared with the Ctrl, as the values obtained in Str were considerably better. Regarding AI and TI, values of 0.6–0.7 can be considered low, despite recommendations of indices lower than 0.5 to fully benefit from the beneficial effects [11]. Both extracts presented high AI and TI values, although the TI index was very close to the acceptable levels in the Ctrl, and close to the desired levels in the Str. Aussant et al. (2018) studied the fatty acid composition of *N*. *oculata* (SAG 38.85) grown under different temperatures, with the objective of obtaining the highest EPA amount [11]. They found, under the optimum conditions for EPA production, an AI of 0.71 and a TI of 0.47, values which were lower than those registered herein. Matos et al. (2016) also determined AI and TI indices for *N*. *oculata* (strain not identified) culture, finding even lower values, namely 0.63 and 0.22, respectively, which were the consequence of a high PUFA percentage, as the culture presented 37% PUFA [36].

Concerning the HH ratio, for which higher values are better, corresponding to a higher decrease in blood cholesterol, as previously mentioned, the Str presented a considerably higher value than did the Ctrl. Compared with the findings of other authors, Matos et al. (2016) obtained a significantly higher HH (1.44), while Aussant et al. (2018), although not determining the same ratio, but rather determining the hypocholesterolemic index (which equation is similar), also found a higher value (0.90) [11,36].

#### 2.1.2. Sterol Profiles and Pigment Contents

The analysis of sterol profiles (Table 2) revealed that the extract with the highest total sterols content was the Ctrl, representing 2.8% of the extract, with 43% more sterols than the Str extract, in which sterols accounted for 1.9% of the total extract. This difference between the extracts was mainly due to cholesterol content, which in the Ctrl was 75% higher; regarding the remaining sterols, Ctrl only presented a significantly different (*p* < 0.05) amount of campesterol (45% higher). Cholesterol was found to be the main component of both extracts, representing 67 and 55% of total sterols in the Ctrl and Str, respectively, followed by β-sitosterol, which accounted for 20 and 30% (0.56 and 0.58% of total lipids), respectively. Presenting lower amounts, and consequently, representing lower percentages, stigmasterol, campesterol, and desmosterol were the remaining sterols found in the extracts.

Concerning pigment contents (Table 2), the extract obtained from the culture grown under modulated temperature stress conditions presented higher pigment contents than the extract originated from cells grown under normal conditions. Higher amounts of all pigments were observed in the Str, which presented (in comparison with Ctrl) 31, 75, and 20% increased contents of Chl *a*, Chl *b*, and carotenoids, respectively. The main pigment found in the extracts was Chl *a*, which accounted for around 60% of the total pigments, while carotenoids composed 37 and 35% of the Ctrl and Str, respectively. Overall, pigments represented 7.5% of the Ctrl and 9.5% of the Str.

The sterol profiles of both extracts are only partially in accordance with those reported in the literature concerning *N*. *oculata*, since most studies found higher sterol percentages than those obtained herein. Mouahid et al. (2013) extracted lipids from *N*. *oculata* and found that sterols represented 1.8% of the extract, similarly to the results for the Str (1.9%) [37]; on the other hand, Crampon et al. (2013) determined *N*. *oculata* sterols content between 4.6 and 6.6% of the extract, depending on the extraction procedure, which is considerably higher than those registered for the Ctrl and Str, respectively [38]. Xu et al. (2012) and Geng et al. (2016) also reported sterols content within the same order of magnitude (6.2 and 6.3%, respectively), and of similar nature, being those fractions composed by cholesterol (89.2 and 88.7%; 5.5 and 5.6% of total lipids), followed by stigmasterol (7 and 7.4%, respectively) [39,40]. In lower percentages, β-sitosterol (1.6 and 1.4%) and campesterol (1.3 and 1.6%) were also registered, as well as brassicasterol and fucosterol, each representing 0.03% of the lipid extract.

Custódio et al. (2012) found even higher contents in hexane extract of *N. oculata*, with cholesterol and fucosterol presenting an 8.6 and 1.3% relative abundance, which together accounted for 9.8% of the extract, and did not detect campesterol which, although at low levels, was found in both extracts in the present study [41]. Sanjeewa et al. (2016) reported a sterol content of 19.38% in a methanolic extract (80% *v*/*v*) of *N. oculata*., and Dunstan et al. (1993) found the *N. oculata* lipid content to be composed by 4-desmethyl sterols, which represented 3.9 to 5.9% of lipids, depending on the growth phase of the culture (logarithmic or stationary) and on the culture technique (batch or semicontinuous) [20,42].

The differences between sterols contents and composition in the Ctrl and Str and those found in the abovementioned studies may result from several circumstances, such as the utilization of different strains of *N*. *oculata*. Moreover, as reported by Dunstan et al. (1993), the growth phase of the culture can also influence the sterol composition of the lipid fraction [42], and growth conditions, such as light intensity and temperature, which were different in each of the mentioned research works, have also been shown to impact sterol concentration in microalgae [43]. The differences may also result from the utilization of different extraction solvents, as several authors [38,39,40,42] used chloroform:methanol (1:2) [44], hexane [41], 80% methanol solution [20], or supercritical CO_2_ [37,38]. The use of extractions solvents with different compositions and polarities influences the type and amount of compounds extracted in the different studies, resulting in different sterol profiles.

Concerning the pigments, the results described herein are also only partially in agreement with those reported in the literature, particularly concerning chlorophylls. Mouahid et al. (2020) extracted pigments from *N*. *salina* and *N*. *maritima* using supercritical CO_2_ with ethanol as a co-solvent, and found Chl *a* and carotenoids (maximum of 0.34 and 0.78 ug/g for *N*. *salina*, and 1.39 and 0.95 ug/g for *N*. *maritima*, respectively) in the extract only [45]. Previously, Nobre et al. (2013) had studied the extraction of pigments from *Nannochloropsis* sp. using the same technology and solvents, and also only reported low amounts of Chl *a* (33.7 mg/g of extract) and several carotenoids [46]. Nonetheless, Parniakov et al. (2015) utilized pulsed electric fields and ultrasounds as adjuvants to pH-assisted aqueous extraction to extract pigments from *Nannochloropsis* spp. and also found Chl *b* to be present in the extracts, despite only reporting total chlorophylls and carotenoids amounts (~20 and 9 mg/g, respectively) [47].

Carotenoid content in *Nannochloropsis* is more widely described across the studies. Besides the abovementioned studies, Millao and Uquiche (2016) extracted carotenoids from pelletized *N. gaditana* using supercritical CO_2_, yielding 4.5 mg/g_oil_, while Maadane et al. (2015) were only able to extract 3.0 mg/g_extract_ from *N. gaditana* biomass, utilizing ethanol [48,49]. Feller et al. (2018) applied supercritical CO_2_ and subcritical n-butane *N. oculata*, and yielded 4.4 and 3.1 mg/g_extract_, respectively [50]. Similarly to sterols, the discrepancy between these results and the higher yields and composition reported herein, may be a consequence of the different species of *Nannochloropsis* analyzed, the different extraction methodologies/solvents applied in the extraction process, or the different culture conditions and growth stages in which biomass was collected for extraction.

### 2.2. Oxygen Radical Absorbance Capacity (L-ORAC_FL_)

Antioxidant capacity was assessed through L-ORAC_FL_, and the results (Table 3) showed no significant difference (*p* > 0.05) between the lipophilic extracts. The absence of a difference between the Ctrl and Str may have resulted from a balance (combination) between the antioxidant capacity of the sterols and that of the pigments (namely, carotenoids), since such activity has been ascribed to both types of compounds [20,50]. In this sense, although the compositions of the extracts were distinct in terms of individual compounds (and compound types), the overall oxygen radical absorbance capacity was not significantly different (*p* > 0.05).

### 2.3. Biological Activities

#### 2.3.1. Metabolic Activity—MTT Assay

**Figure 1:**
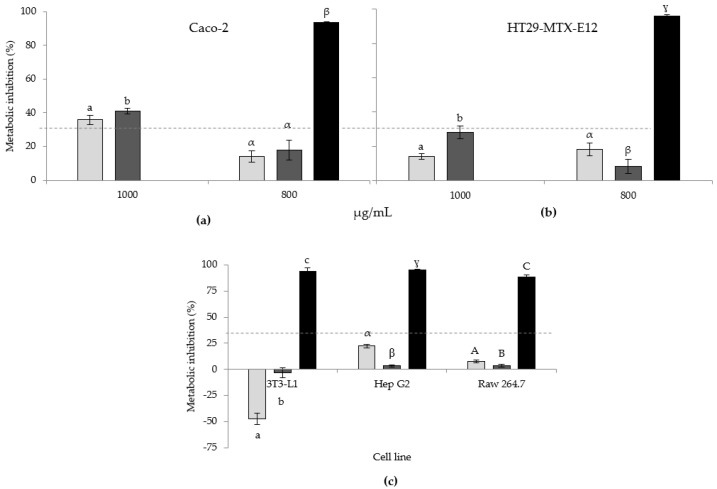
Metabolic inhibition of the distinct cell lines when in contact with dimethyl sulfoxide (DMSO; control (■)), Ctrl (■), or Str (■) extracts. The dashed line (--) indicates the 30% significant inhibition threshold. In (**a**,**b**), extracts at 1000 and 800 µg/mL are shown, and in (**c**), extracts solely at 800 µg/mL are shown. Results are expressed as average ± standard deviation (*n* = 3). Within each individual cell line, and within each concentration, values with the same letter are not significantly different (*p* > 0.05).

The cytotoxicity of the extracts was evaluated concerning the Caco-2, HT29-MTX-E12, Hep G2, 3T3-L1, and RAW 264.7 cell lines using the MTT cell metabolic activity assay. Figure 1a,b portrays the impact of the extracts on human colon epithelial Caco-2 and HT29-MTX-E12 cell lines at 1000 and 800 µg/mL. Concerning the Caco-2 cell line (Figure 1a), at the highest concentration, both extracts present metabolic inhibition above the threshold (30%) considered as significant (as defined in ISO 10993-5:2009 [51]), meaning that at 1000 µg/mL, the extracts were detrimental to the normal metabolic activity of those cells, while at 800 µg/mL, the inhibition was considerably lower (<18%), below the required limit, and therefore, not regarded as significant. Regarding the HT29-MTX-E12 cells (Figure 1b), the metabolic inhibition at 1000 µg/mL was lower than 30%; however, the Str presented a close value of 28.2%, with the standard deviation rising above the threshold.

As potential applications of the extracts might involve their incorporation in food products, since a significant inhibition of metabolic activity of colon epithelial cell lines was observed at 1000 µg/mL, which was absent at 800 µg/mL, it was determined that in the remaining assays, the extracts would be tested solely at 800 µg/mL.

The results pertaining to the assessment of the metabolic inhibition of the remaining cell lines with the extracts at 800 µg/mL are presented in Figure 1c, showing no significant inhibition of any of the cell lines. In fact, contrastingly, both extracts increased the metabolic activity of mouse pre-adipocytes 3T3-L1. Although Str only increased metabolic activity very slightly (3.4%), when in contact with the Ctrl, mouse pre-adipocytes metabolism was increased by 47.4%. The observed impact of the extracts on the 3T3-L1 cell line may be the result of the lipid content of the extracts, since the cells are pre-adipocytes, which increase metabolic activity when in contact with lipids. Such a rationale may explain the higher increase observed regarding the Ctrl, in comparison with the Str, since the lipid content is higher (455.5 and 398.3 mg_fatty acid_/g_extract_, respectively).

The results obtained herein indicated that, due to the absence of significant metabolic inhibition, the lipid extracts may be utilized (up to 800 µg/mL) in the development of products that may be safely consumed without causing detrimental cellular metabolic alterations. Although no reports were found in the literature in which *N*. *oculata* lipid extracts were assessed regarding metabolic inhibition of the abovementioned cell lines, from the perspective of anticarcinogenic potential, Atasever-Arslan et al. (2016) performed a methanolic extraction of the microalga biomass, testing cytotoxicity against HL60 (human promyelocytic leukemia cell line), K562 (human chronic myeloid leukemia cell line), and ECV304 (human umbilical vein endothelial cell line) [52]. The study found the toxicity of the extract (>30%; i.e., cell viability < 70%) at 1 µg/mL in K562 cells and 500 µg/mL in HL60, the latter being closer to the levels observed herein pertaining to Caco-2 cells. At the concentrations tested, no cytotoxicity was registered in the non-cancerous ECV304 cell line. Custódio et al. (2015) utilized hexane, diethyl ether, acetone, and water to obtain *N*. *oculata* extracts, which were assessed for toxicity in a human neuroblastoma cell line (SH-SY5Y) [18]. It was found that cell viability decreased more than 20% at concentrations higher than 50 µg/mL, which is an amount nearly 20-fold lower than that determined in the present study to attain a similar impact on Caco-2 cells. More recently, Wali et al. (2020) studied the anti-cancer activity of an *N*. *oculata* methanolic extract, which was found to inhibit cell viability by 25% at 200 µg/mL in the MDA-MB-231 human breast cancer cell line, which is 5 times lower than the 1000 µg/mL reported herein concerning Caco-2 [21].

#### 2.3.2. Adipolysis Assay

Adipolysis is a highly regulated process which enables the necessary delivery of free fatty acids to meet energy requirements, and it is related to the degradation of the triglycerides in adipose tissue [53]. In the current assay, isoproterenol was utilized as the positive control for the triglyceride degradation inside the cell, resulting in an increase in the glycerol concentration in the culture media. Due to the conversion of triglycerides into free fatty acids and glycerol, it is possible to determine the degree of adipolysis by measurement of the concentration of glycerol released [54].

**Figure 2:**
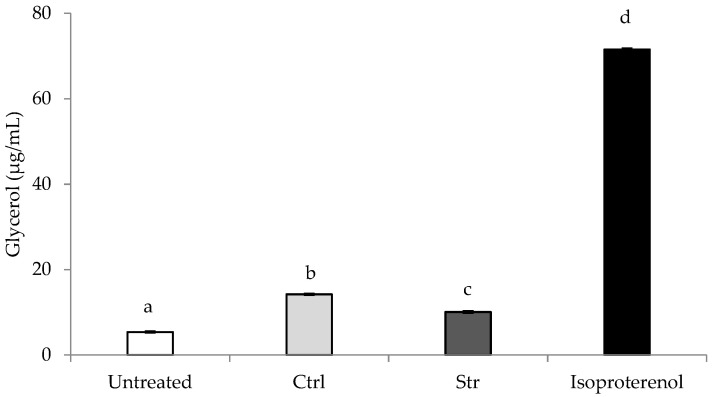
Glycerol released by 3T3-L1 differentiated adipocytes in the adipolysis assay. Results are expressed as average ± standard deviation (*n* = 3). Values with the same letter are not significantly different (*p* > 0.05).

Compared with the control (non-treated cells), the results (Figure 2) revealed a significant (*p* < 0.05) increase in glycerol release by the 3T3-L1 differentiated adipocytes when in the presence of both extracts. The non-treated cells released 5.4 ± 0.2 µg/mL of glycerol, while cells treated with lipid extracts released 14.2 ± 0.2 and 10.1 ± 0.3 µg/mL (Ctrl and Str, respectively).

The adipolysis observed in the presence of the extracts is in accordance with the MUFA and ω-3 PUFA contents of the extracts, since those fatty acids are reported to induce adipolysis, through several mechanisms, the former (e.g., oleic acid) by the activation of peroxisome proliferator-activated receptor-α (PPAR-α), and the latter (e.g., EPA) by the promotion of the β-oxidation of fatty acids in adipose tissue via the activation of 5′ adenosine monophosphate-activated protein kinase (AMPK) [55,56,57]. Moreover, EPA also promotes mitochondrial biogenesis and consequently, an increase in energy metabolism. EPA inhibition of lipogenesis and increases in fatty acid oxidation may be responsible for anti-obesity effects [56,57,58]. Concerning the difference between extracts, it may be explained due to the higher MUFA content of the Ctrl, in comparison with the Str (181.6 and 158.7 mg_fatty acid_/g_extract_, respectively), which resulted in a higher degree of adipolysis, despite the higher EPA content of the latter.

#### 2.3.3. Hepatic Lipid Accumulation

Non-alcoholic fatty liver disease (NAFLD) can be characterized by distinct stages of liver injury, namely hepatic steatosis (triglyceride accumulation) as the first stage, which can evolve into non-alcoholic steatohepatitis (NASH) and may eventually result in cryptogenic cirrhosis or hepatocellular carcinoma [59,60], and the development of NAFLD has been ascribed to changes in dietary fatty acid intake. In obese NAFLD patients, the high saturated/unsaturated fatty acids ratio and the high free fatty acids levels are correlated with insulin resistance, which is regarded as a main determinant in NAFLD pathogenesis [61,62]. Despite the fact that hepatocytes are not the physiological site of lipid storage, steatosis development is linked to cellular dysfunction and apoptosis [59,62].

**Figure 3:**
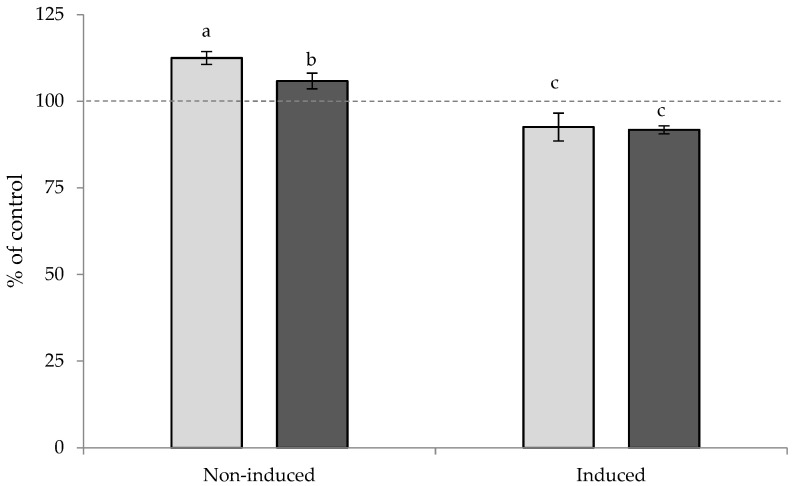
Hepatic lipid accumulation of non- and steatosis-induced Hep G2 hepatocytes, when in contact with the Ctrl (■) or Str (■). Results are expressed as average ± standard deviation (*n* = 3). Values with the same letter are not significantly different (*p* > 0.05).

Lipid accumulation was assessed in non- and steatosis-induced (with chloroquine) Hep G2 hepatocytes, treated with microalga lipid extracts at 800 µg/mL. The results (Figure 3) showed that both extracts induced lipid accumulation in steatosis non-induced (normal) cells, to a higher extent than in the control (steatosis-induced), with Ctrl accounting for the highest percentage (112.5 and 105.8% of control, respectively). However, in steatosis-induced cells, the extracts were able to decrease hepatic lipid accumulation to 92.5 and 91.7% of the control (Ctrl and Str, respectively).

The results obtained herein may be related to the palmitic and oleic acids present in the extracts, both of which are known to be steatosis inducers. Although oleic acid is reported to be more steatogenic than palmitic acid—and more easily oxidized—palmitic acid induces a higher extent of apoptosis [55,62]. Concerning the results presented herein, the combination of the apoptosis resulting from the palmitic acid, with the oxidation of the oleic acid, surpassed the steatogenesis originated by the oleic acid and, therefore, steatosis was more pronounced when the Hep G2 cells were in the presence of the Ctrl. Moreover, the saturated to unsaturated fatty acid (SFA/UFA) and omega-6 to omega-3 (ω6/ω3 or n6/n3) ratios (0.72 and 0.64, for Ctrl and Str, respectively) have also been associated with lipid accumulation steatosis, with higher values being correlated with increased steatosis [55,57]. Cholesterol is also known to induce hepatic lipid accumulation [63] and therefore, must have also played a role in lipid accumulation, which is in accordance with the results, since the highest steatosis was observed in the Ctrl extract, which presented the highest cholesterol content.

The contrasting “anti”-steatosis impact of the extracts in steatosis-induced cells may be ascribed to the EPA contents, since that PUFA was reported to reduce hepatic forkhead box protein O1 (FoxO1), which indicated gluconeogenesis inhibition [64]. EPA was also shown to decrease hepatic triglycerides content/accumulation via the downregulation of sterol regulatory element binding protein-1c (SREBP-1c), which decreases the transcription of lipogenic genes [65]. Hence, the levels of the encoded lipogenic key enzymes, such as PPARs and fatty acid synthase (FAS), among others, are also decreased [64,65].

#### 2.3.4. Anti-Inflammatory Activity

Inflammation is a process by which an organism defends itself against an infection or an insult, and it has the roles of attacking pathogens, repairing damaged tissues, and restoring homeostasis [14,66]. Among the mechanisms comprised in inflammatory response to a stimulus is the activation of several cytokines, some of which have a pro-inflammatory activity, e.g., interleukin-6 (IL-6) and interferon-β (IFN-β), while others, such as interleukin-10 (IL-10), are anti-inflammatory, in order to control the inflammation so that it does not become harmful to the host [66,67]. Despite the beneficial effects of those cytokines in the inflammatory process, there is also the potential for a detrimental impact on the host when there is a deregulation of expression. Imbalanced (over or under) secretions of IL-6 and IL-10 are reported to originate several inflammatory and autoimmune diseases, such as rheumatoid arthritis and diabetes mellitus [67,68].

The anti-inflammatory activity of the *N. oculata* extracts was assessed through measurement of the amounts of distinct cytokines, namely, IL-6, IL-10, and IFN-β, expressed by RAW 264.7 macrophages in both basal metabolism, as well as under lipopolysaccharide (LPS) stimulus.

**Figure 4:**
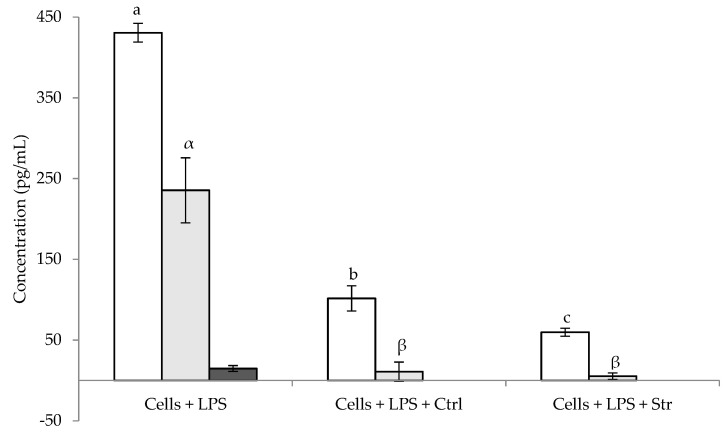
Cytokine expression in LPS-stimulated RAW 264.7: IL-6 (□), IL-10 (■), and IFN-β (■). Results are expressed as average ± standard deviation (*n* = 3). Within each individual cytokine, values with the same letter are not significantly different (*p* > 0.05).

The determination of the concentration of the cytokines in non-stimulated cells revealed no production of those compounds, neither in the control cell, nor in cells exposed to either of the extracts. Contrastingly, when stimulated by LPS (Figure 4), as expected, the control cells secreted all the assessed cytokines, while the presence of the extracts led to decreased amounts thereof. Concerning the impact of the extracts on the expression of the inflammatory markers, cells in contact with the Ctrl presented lower amounts than those found in the control (cells + LPS), and when exposed to the Str, the decrease was even higher, registering the lowest concentrations.

The results observed in LPS-stimulated RAW 264.7 macrophages are only partially in agreement with those found in the literature, particularly because of the different conditions employed (such as microalga species, cell lines, and inflammation markers) compared to those utilized herein. Nonetheless, a somewhat similar study was performed by Nacer et al. (2020) [69], who evaluated the impact of feeding *N*. *gaditana* to diabetic rats which, compared with controls, presented increased IL-6 serum levels. The study found lower levels of IL-6 in diabetic rats fed with an *N*. *gaditana* containing diet, which indicated an anti-inflammatory activity of the microalga, since IL-6, as previously mentioned, is a pro-inflammatory interleukin; these results are in accordance with the results obtained herein. The anti-inflammatory effect of a sterol-rich fraction of *N*. *oculata* was demonstrated by Sanjeewa et al. (2016) [20], who utilized LPS-stimulated RAW 264.7 macrophages. However, the anti-inflammatory activity was determined through the downregulation of the inducible nitric oxide synthase (iNOS) and cyclooxygenase-2 (COX-2) proteins, which are stimulated in an inflammation context. Contrastingly, there are studies which ascribe a positive effect of *N*. *oculata* to the immune system of the host, in response to an inflammatory stimulus. Revianti et al. (2020) orally administered *N*. *oculata* to rats infected with a periodontopathic bacterium to assess the anti-inflammatory activity of the microalga [70]. As expected, the study revealed a higher expression of IL-10 than that observed in the control when the animals were subjected to an inflammatory stimulus (infection), an expression which was increased when the animals were orally irrigated with a solution of the microalga (ca. 2.5% *w*/*v*). The pro-inflammatory tumor necrosis factor-α (TNF-α) was also quantified and observed to be decreased in microalga-administered infected rats. Abdelghany et al. (2020) [71] studied the effect of dietary supplementation of Nile tilapia (*Oreochromis niloticus*) with *N*. *oculata* in the expression of hepatic pro-inflammatory cytokines regulator genes, finding an upregulation in the animals which were fed *N*. *oculata*.

The anti-inflammatory activity observed in the present study regarding IL-6 and IFN-β is in agreement with those reported concerning omega-3 and omega-6 PUFA. Omega-6 PUFA are known to be pro-inflammatory, and omega-3 PUFA are known to reduce inflammation, being of an anti-inflammatory nature [12,13,14], which was observed herein, since the decrease in the expression of the pro-inflammatory cytokines was the highest in the LPS-induced cells exposed to the extract with higher omega-3 PUFA (Str).

## 3. Materials and Methods

### 3.1. Microalgae and Growth Conditions

**Figure 5:**
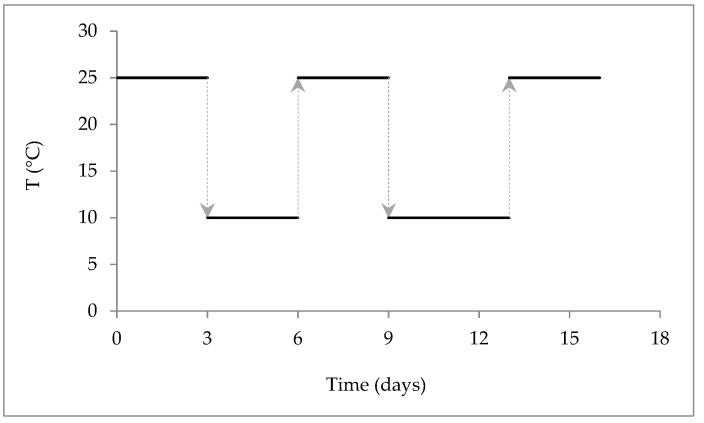
Temperature stress modulation throughout growth.

The microalga used in the study was *Nannochloropsis oculata* CCAP 849/1, (SAMS Ltd., Scottish Marine Institute, Scotland, UK). The culture was grown in modified artificial seawater medium (ASW) [72] for 16 days, under two temperature regimens: one isothermal at 25 °C, which served as the control, and one in which the temperature was modulated throughout growth (Figure 5) to stress the cells in a modulated fashion, thus increasing the EPA concentration without decreasing cell growth, according to the method described by Sousa et al. (2022) [30]. The cultures were designated as Ctrl and Str, respectively. Both were grown under a continuous light intensity of 75 µmol photons/m^2^/s, provided by cool daylight fluorescent lamps (Lumilux L18W/865, OSRAM, Munich, Germany), in a climate chamber S600PL (Aralab, Rio de Mouro, Portugal).

### 3.2. Lipid Extraction and Characterization

#### 3.2.1. Lipid Extraction

Microalga biomass from the two cultures was collected by centrifugation at 1400× *g* and 4 °C, for 5 min. Supernatants were discarded, pellets were added the extraction mixture (diethyl ether:ethanol (Et_2_O:EtOH, 2:1)) in a 1:5 (mL/mg_AFDW_) ratio, and placed in a water bath at 40 °C for 15 min. Afterwards, solvent partition was performed with water at a 2:1 ratio by vortexing and subsequently centrifuging the solution; the upper layer containing lipophilic compounds was then collected. Diethyl ether was evaporated with a rotary evaporator, the lipid content was determined by gravimetry, and the solution was resuspended in Et_2_O and stored at −20 °C. When the extracts were used in the different methodologies, a certain amount (volume) of extract was evaporated under nitrogen and the weight was determined by gravimetry, after which it was resuspended in the appropriate solvent for the specific assay. The lipid extracts were designated as Ctrl and Str.

#### 3.2.2. Lipid Characterization

##### Fatty Acid Profiles

Fatty acid profiles were determined by gas chromatography using a flame ionization detector (GC-FID), according to the protocols described by Sousa et al. (2022) [30].

Furthermore, concerning lipid quality, two indices and one ratio, namely the atherogenic index (AI), the thrombogenic index (TI), and the hypocholesterolemic: hypercholesterolemic ratio (HH), were calculated according to Mitra and Mishra (2019), using Equation (1) [73].

(1a) $$AI=\frac{\left[C12:0+4\times \left(C14:0\right)+C16:0\right]}{\left(\sum MUFA+\sum PUFA\;\omega 6+\sum PUFA\;\omega 3\right)}$$

(1b) $$TI=\frac{\left(C14:0+C16:0+C18:0\right)}{\left[0.5\times \sum MUFA+0.5\times \sum PUFA\;\omega 6+3\times \sum PUFA\;\omega 3+\left(\frac{\sum PUFA\;\omega 3}{\sum PUFA\;\omega 6}\right)\right]}$$

(1c) $$HH=\frac{\left(C18:1\omega 9+C18:2\omega 6+C18:3\omega 3+C20:4\omega 6+C20:5\omega 3\right)}{\left(C14:0+C16:0\right)}$$

##### Sterol Profiles

Twenty milligrams of lipid extract were dried under nitrogen, after which 100 µL of 5α-cholestan-3β-ol (internal standard; Sigma-Aldrich, St. Louis, MO, USA) were added, followed by 1.8 mL of methanol and 200 µL of sodium methoxide (5.4 M; Acros Organics, Geel, Belgium), and the mixture was heated at 90 °C for 15 min. After rapid cooling (ice), 1.5 mL of sulfuric acid (3 M) in methanol were added, and the mixture was reheated at 90 °C for 2 min. After cooling down, 5 mL of saturated sodium chloride solution and 2 mL of hexane were added, and the samples were vortexed and centrifuged at 1250× *g* for 10 min. The resulting upper layer (hexane) was collected for a new glass tube and evaporated to dryness under nitrogen. Afterwards, the derivatization to trimethylsilyl ether derivatives was performed by adding 500 µL of acetonitrile and 100 µL of N,O-Bis(trimethylsilyl)trifluoroacetamide (BSTFA; Sigma-Aldrich, St. Louis, MO, USA) and heating at 70 °C for 30 min. Acetonitrile was then evaporated to dryness under nitrogen, and the samples were resuspended in 300 µL of hexane.

The samples were analyzed by GC-FID (CLARUS 500 model, Perkin Elmer, Waltham, MA, USA) equipped with a VF5ms column (30 m × 0.25 mm × 0.25 μm; Agilent Technologies, Sta Clara, CA, USA). The analysis conditions were selected according to the methods of Salta et al. (2008), with slight modifications: helium was used as the carrier gas at a flow rate of 19.2 mL/min. The injector and detector temperatures were 140 and 290 °C, respectively [74]. The split was 30:1, and the injection volume was 1 µL. The oven temperature was initially set at 100 °C and then increased at a rate of 20 °C/min to a final temperature of 300 °C, at which it remained for 15 min. The identification of sterols was performed using a plant sterol mix (Larodan, Solna, Sweden) and individual standards.

##### Pigment Content

The pigment content was assessed according to the methodology described by Lichtenthaler and Buschmann (2005), with the extract resuspended in Et_2_O. Extract absorbance at different wavelengths was measured, and the chlorophyll a (Chl *a* (*c_a_*)), chlorophyll b (Chl *b* (*c_b_*)), and carotenoids (*c*_car_) contents were determined using Equation (2) [75].

(2a) $$C_{a}\;\left(µg/\mathrm{mL}\right)=10.05\;Abs_{661}-0.97\;Abs_{642}\;$$

(2b) $$C_{b}\;\left(µg/\mathrm{mL}\right)=16.36\;Abs_{642}-2.43\;Abs_{661}\;$$

(2c) $$C_{car}\;\left(µg/\mathrm{mL}\right)=\left(1000\;Abs_{470}-1.43\;c_{a}-35.87\;c_{b}\right)/205\;$$

### 3.3. Oxygen Radical Absorbance Capacity (ORAC_FL_)

Antioxidant capacity was determined using an ORAC_FL_ assay. The lipid extracts were analyzed with lipophilic ORAC (L-ORAC_FL_), according to the method of Poyato et al. (2013), with slight changes [76]. Briefly, 5 mg of extract were resuspended in 400 µL of an ethanol:acetone (7:3, *v*/*v*) solution, and 4.6 mL of a 7% (*w*/*v*) randomly methylated *β*-cyclodextrin (RMCD; Cyclolab, Budapest, Hungary) solution (1:1, acetone:water, *v*/*v*) were added. The mixture was then shaken at 800 rpm in an orbital shaker for 1 h at room temperature. Trolox (6-hydroxy-2,5,7,8-tetramethylchroman-2-carboxylic acid; Sigma-Aldrich, St. Louis, MO, USA) stock solution (1 mM) was prepared in a phosphate buffer solution (PBS; 75 mM, pH 7.4) and stored at −20 °C. When the assays were performed, an initial Trolox solution of 100 µM was prepared with RMCD and further diluted with PBS to obtain standards from 10–80 µM. RMCD was also used to dilute samples and as a blank. The assays were performed in 96-well U bottom, polypropylene black microplates (Thermo Scientific^TM^, Nunc^TM^, Roskilde, Denmark), with 20 µL of sample (standards or blank), to which 120 µL of fluorescein (FL, 116.66 nM; Sigma-Aldrich, St. Louis, MO, USA) were added, and equilibrated for 10 min at 37 °C. Afterwards, the reaction was initiated by the addition of 60 µL of 2,2′-azobis(2-methylpropionamidine) dihydrochloride (AAPH, 48 mM; Sigma-Aldrich, St. Louis, MO, USA), and immediately read (Synergy H1, Biotek Instruments, Winooski, VT, USA), throughout 80 min, at 1 min intervals. The results were expressed as µmol Trolox equivalent/mg (extract).

### 3.4. Biological Activities

#### 3.4.1. Cell Lines Growth Conditions

Human Caucasian colon adenocarcinoma epithelial cells and human colon mucous-secreting epithelial cells were obtained from the European Collection of Authenticated Cell Cultures (ECACC, Porton Down, UK), Caco-2 (ECACC 86010202), and HT29-MTX-E12 (ECACC 12040401), respectively. Human epithelial-like hepatocellular carcinoma Hep G2 (American Type Culture Collection, ATCC; ATCC HB-8065), mouse pre-adipocytes 3T3-L1 (ATCC CL-173), and mouse macrophages RAW 264.7 (ATCC^®^ TIB-71™) were purchased from ATCC (Manassas, VA, USA). Caco-2, HT29-MTX-E12, Hep G2, and RAW 264.7 cells were cultured at 37 °C in a humidified atmosphere of 95% air and 5% CO_2_ as monolayers, using Dulbecco’s Modified Eagle’s Medium (DMEM) with 4.5 g/L glucose, L-glutamine without pyruvate (Gibco, Thermo Scientific, Waltham, MA, USA), containing 10% (*v*/*v*) of fetal bovine serum (FBS, Biowest, France). The culture medium of Caco-2 and HT29-MTX-E12 cells was further supplemented with 1% (*v*/*v*) of non-essential amino acids (Gibco, Thermo Scientific, Waltham, MA, USA). Pre-adipocytes (3T3-Ll) were cultured in DMEM with 10% (*v*/*v*) of iron-fortified calf bovine serum (ATCC 30-203, Manassas, VA, USA) and 1% (*v*/*v*) of penicillin-streptomycin-fungizone (Lonza, Basel, Belgium).

#### 3.4.2. Extracts Preparation for Cell Culture Assays

Lipid extracts of *Nannochloropsis oculata* were dried under nitrogen and resuspended in ethanol. The initial extract was diluted 100 times in DMEM to achieve final ethanol and extract concentrations of 1% (*v*/*v*) and 1 mg/mL, respectively. The subsequent dilutions, to obtain extract concentrations of 800, 400, 200 and 100 µg/mL, were also performed in DMEM with 1% (*v*/*v*) ethanol.

#### 3.4.3. MTT Assay

The MTT assay was performed according to the method described by Mosmann (1983) and the international standard ISO 10993-5 [51,77]. The percentage of metabolic inhibition was calculated using Equation (3), and was only considered significant if it was above 30%, according to the international standard [51].

(3) $$Metabolic\;Inhibition\;\left(\%\right)=\frac{Abs_{positive\;control}-Abs_{sample}}{Abs_{positive\;control}}\times 100$$

#### 3.4.4. Adipolysis Assay

The adipolysis assay was performed in mouse pre-adipocytes 3T3-L1 (ATCC CL-173) using the Adipolysis Assay Kit (Abcam ab133115; Abcam, Cambridge, UK), according to the manufacturer’s instructions, using isoproterenol solution (10 µM) as a positive control and cell culture medium as a negative control. The degree of adipolysis was determined through the concentration of glycerol released.

#### 3.4.5. Hepatic Lipid Accumulation

The hepatic lipid accumulation assay was performed using the hepatocellular carcinoma Hep G2 cell line, with the Hepatic Lipid Accumulation Kit (Abcam ab133131; Abcam, Cambridge, UK), according to the manufacturer’s instructions. Briefly, the hepatocytes were seeded at a density of 10^4^ cells/mL in a 96-well plate; after 24 h, the medium was removed and replaced by fresh medium, with samples diluted 1:100, or steatosis induction control (chloroquine 25 μM). After 72 h of exposure, the cells were stained with Oil Red O and examined by measuring the absorbance at 490 nm in a microplate reader (Synergy H1). To evaluate the possible effect of fatty acids on hepatic steatosis, one additional assay was conducted, in which test samples were diluted in medium with chloroquine 25 µM.

#### 3.4.6. Anti-Inflammatory Activity

The anti-inflammatory activity of the extracts was assessed in mouse macrophages RAW 264.7 (ATCC^®^ TIB-71™) using BioLegend’s LEGENDplex^TM^ 740446 mouse inflammation panel immunoassay (BioLegend, San Diego, CA, USA), according to the manufacturer’s instructions.

### 3.5. Statistical Analysis

Statistical analysis was performed using SPSS^®^ statistics software (IBM^®^, Chicago, IL, USA), with analysis of variance (ANOVA) being used to determine differences between three groups, with Tukey’s as post hoc test. When the assay was only constituted by two groups (namely, Ctrl and Str), the *t*-test was utilized to assess statistical differences. All tests were performed using a 0.05 significance.

## 4. Conclusions

Modulated temperature stress impacted *N*. *oculata* lipid composition, increasing EPA and pigment contents, concomitantly decreasing sterols’ (including cholesterol). The changes in the composition of the lipophilic extract obtained from microalga growth under modulated stress positively impacted the assessed biological activities, which displayed, when compared with the control, increased anti-steatosis and anti-inflammatory activities.

Overall, the results revealed that growth under modulated temperature stress enhanced *N. oculata* lipid-associated health effects, and that lipid extracts may be considered safe for consumption, since no inhibition of metabolic activity of the evaluated cell lines was observed, which indicates that those extracts can be incorporated in formulations to be used in the development of novel food products.

## Figures and Tables

**Table 1:** Fatty acid profiles of extracts (mg_fatty acid_/g_extract_).

Fatty Acid	Ctrl	Str	Fatty Acid	Ctrl	Str
C12:0	2.32 ± 0.06 ^a^	1.95 ± 0.01 ^b^	α C18:3 c9c12c15	0.57 ± 0.12 ^a^	0.38 ± 0.01 ^b^
C14:0	16.65 ± 0.39 ^a^	17.73 ± 0.22 ^b^	C20:0	0.98 ± 0.13 ^a^	0.89 ± 0.01 ^a^
C14:1	0.48 ± 0.01 ^a^	0.40 ± 0.01 ^b^	C20:1 c9	0.18 ± 0.01 ^a^	0.20 ± 0.01 ^a^
C15:0	3.87 ± 0.18 ^a^	3.31 ± 0.01 ^b^	C20:2 c11c14	1.07 ± 0.01 ^a^	0.70 ± 0.07 ^b^
C15:1	0.77 ± 0.05 ^a^	0.62 ± 0.01 ^b^	C20:3 n6	1.78 ± 0.02 ^a^	1.07 ± 0.02 ^b^
C16:0	159.41 ± 3.59 ^a^	124.13 ± 1.55 ^b^	C20:4 n6 (AA)	8.93 ± 0.15 ^a^	13.00 ± 0.12 ^b^
C16:1 t9	2.35 ± 0.04 ^a^	1.86 ± 0.08 ^b^	C22:0	0.22 ± 0.01 ^a^	0.20 ± 0.02 ^a^
C16:1 c7	0.43 ± 0.03 ^a^	0.55 ± 0.04 ^b^	C22:1 n11	0.13 ± 0.02 ^a^	0.16 ± 0.02 ^a^
C16:1 c9	158.71 ± 3.53 ^a^	132.38 ± 1.26 ^b^	C20:5 n3 (EPA)	45.25 ± 0.91 ^a^	51.07 ± 0.39 ^b^
C16:1 c11	0.74 ± 0.10 ^a^	0.48 ± 0.08 ^b^	TFA	455.54 ± 11.08 ^a^	398.29 ± 3.58 ^a^
C17:0	1.83 ± 0.16 ^a^	1.82 ± 0.04 ^a^	∑SFA	190.72 ± 4.99 ^a^	155.26 ± 1.67 ^b^
C17:1 c10	1.53 ± 0.19 ^a^	1.53 ± 0.09 ^a^	∑MUFA	181.64 ± 5.04 ^a^	158.69 ± 1.29 ^b^
C18:0 i	0.54 ± 0.12 ^a^	0.58 ± 0.08 ^a^	∑PUFA	83.18 ± 1.05 ^a^	84.34 ± 0.61 ^a^
C18:0	4.90 ± 0.36 ^a^	4.64 ± 0.01 ^a^	∑ω3	45.82 ± 1.03 ^a^	51.45 ± 0.38 ^b^
C18:1 t9	0.71 ± 0.20 ^a^	0.60 ± 0.02 ^a^	∑ω6	37.36 ± 0.02 ^a^	32.89 ± 0.23 ^b^
C18:1 c9	12.70 ± 0.58 ^a^	17.38 ± 0.12 ^b^	ω6/ω3	0.82 ± 0.02 ^a^	0.64 ± 0.01 ^b^
C18:1 c11	2.91 ± 0.30 ^a^	2.53 ± 0.04 ^b^	AI	0.86 ± 0.01 ^a^	0.81 ± 0.01 ^b^
C18:2 c9t12	0.37 ± 0.07 ^a^	0.21 ± 0.03 ^b^	TI	0.73 ± 0.02 ^a^	0.58 ± 0.02 ^b^
C18:2 c9c12	24.80 ± 0.70 ^a^	17.51 ± 0.13 ^b^	HH	0.53 ± 0.02 ^a^	0.71 ± 0.02 ^b^
ɣ C18:3 c6c9c12	0.41 ± 0.10 ^a^	0.40 ± 0.01 ^a^			

Note: Results are expressed as average ± standard deviation (*n* = 3). Values with the same superscript letter are not significantly different (*p* > 0.05). TFA—total fatty acids; SFA—saturated fatty acids; MUFA—monounsaturated fatty acids; PUFA—polyunsaturated fatty acids; AI—atherogenic index; TI—thrombogenic index; HH—hypocholesterolemic:hypercholesterolemic ratio.

**Table 2:** Sterol profiles and pigment contents of the extracts (mg/g_extract_).

Sterol	Ctrl	Str
Cholesterol	18.50 ± 0.22 ^a^	10.04 ± 0.03 ^b^
Desmosterol	0.60 ± 0.10 ^a^	0.56 ± 0.17 ^a^
Campesterol	1.06 ± 0.10 ^a^	0.73 ± 0.10 ^b^
Stigmasterol	2.03 ± 0.27 ^a^	1.68 ± 0.17 ^a^
β-Sitosterol	5.55 ± 0.10 ^a^	5.83 ± 0.48 ^a^
Total	27.75 ± 0.14 ^a^	19.35 ± 0.89 ^b^
**Pigment**		
*c_a_*	45.95 ± 1.12 ^a^	60.18 ± 0.89 ^b^
*c_b_*	1.38 ± 0.06 ^a^	2.41 ± 0.10 ^b^
*c*_car_	27.62 ± 0.98 ^a^	33.25 ± 1.34 ^b^
Total	74.95 ± 3.26 ^a^	95.84 ± 4.71 ^b^

Note: Results are expressed as average ± standard deviation (*n* = 3). Values with the same superscript letter are not significantly different (*p* > 0.05); *c_a_*—chlorophyll *a*; *c_b_*—chlorophyll *b*; *c_car_*—carotenoids.

**Table 3:** Antioxidant activity determined by L-ORAC_FL_.

Sample	µmol_Trolox equivalent_/mg_extract_
Ctrl	49.84 ± 1.46 ^a^
Str	48.54 ± 0.47 ^a^

Note: Results are expressed as average ± standard deviation (*n* = 3). Values with the same superscript letter are not significantly different (*p* > 0.05).

## Data Availability

The data used to support the findings of this study are available from the corresponding author upon request.
